# Identification of 146 Metagenome-assembled Genomes from the Rumen Microbiome of Cattle in Japan

**DOI:** 10.1264/jsme2.ME22039

**Published:** 2022-10-21

**Authors:** Yoshiaki Sato, Hiroaki Takebe, Kazato Oishi, Jumpei Yasuda, Hajime Kumagai, Hiroyuki Hirooka, Takashi Yoshida

**Affiliations:** 1 Department of Agrobiology and Bioresources, School of Agriculture, Utsunomiya University, Tochigi, Japan; 2 Laboratory of Animal Husbandry Resources, Division of Applied Biosciences, Graduate School of Agriculture, Kyoto University, Kyoto, Japan; 3 Laboratory of Marine Microbiology, Division of Applied Biosciences, Graduate School of Agriculture, Kyoto University, Kyoto, Japan; 4 Iwate Agricultural Research Center Animal Industry Research Institute, Iwate, Japan

**Keywords:** Japanese cattle, microbiome, metagenomics, metagenome-assembled genomes, rumen

## Abstract

The rumen contains a complex microbial ecosystem that degrades plant materials, such as cellulose and hemicellulose. We herein reconstructed 146 nonredundant, rumen-specific metagenome-assembled genomes (MAGs), with ≥50% completeness and <10% contamination, from cattle in Japan. The majority of MAGs were potentially novel strains, encoding various enzymes related to plant biomass degradation and volatile fatty acid production. The MAGs identified in the present study may be valuable resources to enhance the resolution of future taxonomical and functional studies based on metagenomes and metatranscriptomes.

The rumen is a forestomach in the ruminant digestive tract that comprises a complex microbial ecosystem of bacteria, ciliate protozoa, fungi, methanogens, and viruses (Morgavi *et al.*, 2013). These microbes degrade plant materials, such as cellulose and hemicellulose (Terry *et al.*, 2019), to yield volatile fatty acids (VFAs), which are a major energy source for ruminants. Therefore, a more detailed understanding the structural and functional characteristics of the rumen microbiome will facilitate enhancements in the efficacy of ruminant production. Recent studies identified several rumen metagenome-assembled genomes (MAGs) (*e.g.*, 4,941 MAGs from Stewart *et al.*, 2019; 1,200 from Wilkinson *et al.*, 2020; and 2,809 from Anderson and Fernando, 2021), which has markedly expanded the rumen microbial database.

Bacterial community compositions in animals appear to exhibit regional variations, which may be because of differences in diet, climate, and farming practices (Henderson *et al.*, 2015). Despite this, we are the only group to have reconstructed rumen MAGs from cattle in Japan. We previously reconstructed 114 rumen MAGs from the shotgun metagenome data of Japanese Black (JB) and F1 crossbred (JB sire×Holstein dam) steers in Japan (Sato *et al.*, 2021). We herein further expanded the catalogs of rumen microbial genomes in Japan with 146 MAGs from the rumen of JB, Japanese Shorthorn (JS), and F1 steers.

The experimental design and protocol were approved by the Kyoto University Animal Ethics Committee (Permit No. R2-119). This study was the first to use JB (*n*=2), JS (*n*=2), and F1 (*n*=6) steers from two farms (for subject data, see Table S1). Rumen contents were collected and processed according to previously reported methods (Sato *et al.*, 2021). To extract DNA, 1.5-mL rumen samples were thawed and centrifuged at 12,000×*g* at 4°C for 15 min. Supernatants were discarded and pellets were treated as previously reported (Frias-Lopez *et al.*, 2008), with modifications from Sato *et al.* (2021). Extracted DNA was stored at –20°C until it was required for further ana­lyses. The metagenomic library was prepared with a Nextera XT DNA library preparation kit (Illumina) and sequenced on an Illumina Hiseq X Ten platform (2×150 bp).

Raw reads in a previous study (Sato *et al.*, 2021; accession number DRA011676) and the present study were trimmed with Trimmomatic version 0.39 (Bolger *et al.*, 2014). To remove host DNA contamination, trimmed reads were mapped with BWA-MEM (Li, H. 2013. Aligning sequence reads, clone sequences and assembly contigs with BWA-MEM. *arXiv.* https://arxiv.org/abs/1303.3997) to the bovine reference genome ARS-UCD1.2/bosTau9. Filtered reads were assembled in SPAdes version 3.13.0 (Bankevich *et al.*, 2012) using the “--meta” option (Nurk *et al.*, 2017), pooled and co-assembled in MEGAHIT version 1.2.9 (Li *et al.*, 2015), then mapped back to contigs with BWA-MEM (Li,‍ ‍H. 2013. Aligning sequence reads, clone sequences and‍ ‍assembly contigs with BWA-MEM. *arXiv.* https://arxiv.org/abs/1303.3997). We binned MAGs with contigs using MetaBAT2 version 2.15 (Kang *et al.*, 2019), MaxBin2 version 2.2.7 (Wu *et al.*, 2016), and CONCOCT version 1.1.0 (Alneberg *et al.*, 2014). Output bins were subsequently dereplicated and aggregated using DAS Tool version 1.1.4 (Sieber *et al.*, 2018). The quality (completeness and contamination) of MAGs was evaluated in CheckM version 1.1.3. (Parks *et al.*, 2015), retaining genomes with ≥50% completeness and <10% contamination. Retained MAGs were dereplicated in dRep version 3.2.2 (Olm *et al.*, 2017) with the “dRep dereplicate” command at 99% and 95% average nucleotide identities (ANI) using a default clustering algorithm, which were hereafter called RUG1_ANI99%_ and RUG1_ANI95%_, respectively, with MAGs in these groups being defined as nonredundant strains and species, respectively (Stewart *et al.*, 2019).

Filtered reads were mapped against RUG1_ANI95%_ MAGs using BamM (https://github.com/Ecogenomics/BamM) to prevent arbitrary mapping between similar genomes (Robbins *et al.*, 2021). Genome relative abundance was calculated using the CoverM version 0.4.0 “genome” (https://github.com/wwood/CoverM) with the following parameters: -m relative_abundance --min-read-percent-identity 0.95 -‍-‍min-read-aligned-percent 0.75. We taxonomically classified RUG1_ANI99%_ MAGs in GTDB-tk version 2.1.1 (Chaumeil, P.A., *et al.* 2022. GTDB-Tk v2: memory friendly classification with the Genome Taxonomy Database. *bioRxiv.* https://doi.org/10.1101/2022.07.11.499641) with GTDB release 207. We then built a phylogenetic tree in PhyloPhlAn version 3.0.60 (Asnicar *et al.*, 2020). The tree was visualized in iTOL version 6.1.2 (Letunic and Bork, 2021). To elucidate whether RUG1_ANI99%_ MAGs represented potential novel species and strains, we compared them with the genomes of rumen prokaryotes (hereafter called RUG2), including Hungate1000 (Seshadri *et al.*, 2018), and previously reported rumen MAGs (Stewart *et al.*, 2019; Anderson and Fernando, 2021) using dRep (Olm *et al.*, 2017). Genomes were defined as novel species (<95%) and strains (<99%) using the ANI outputs by dRep and GTDB-tk.

We predicted the proteins encoded by RUG1_ANI99%_ MAGs with Prodigal version 2.6.3 (Hyatt *et al.*, 2010), and then searched them against the Kyoto Encyclopedia of Genes and Genomes (KEGG) database through GhostKOALA (Kanehisa *et al.*, 2016). Carbohydrate-active enzyme (CAZyme) families, which are associated with cell wall degradation and essential for efficient lignocellulose processing in ruminants, were annotated using dbCAN2 (Zhang *et al.*, 2018). This software integrates three tools (HMMER, DIAMOND, and Hotpep), and we only retained the CAZyme domains annotated by at least two of the three. The predicted protein sequences were then mapped to the Pfam database (Finn *et al.*, 2016) using HMMER version 3.3.2 (Mistry *et al.*, 2013) with an E value <10^–5^ to search cohesin (PF00963) and dockerin (PF00404) domains. We used PULpy (Stewart, R.D., *et al.* 2018 Open prediction of polysaccharide utilisation loci (PUL) in 5,414 public *Bacteroidetes* genomes using PULpy. *bioRxiv.* https://doi.org/10.1101/421024) to predict polysaccharide utilization loci (PUL), linked gene clusters that encode the cell envelope-associated enzymes required for sensing, binding, and degrading polysaccharide substrates (Bjursell *et al.*, 2006). All novel sequence raw data were deposited in DDBJ (accession number DRA014084). The 146 RUG1_ANI99%_ MAGs are available at https://doi.org/10.6084/m9.figshare.20705449.

Filtered samples yielded 706 M reads, and we retained 291 bins. Of these, 202 MAGs had ≥50% completeness and <10% contamination, with 54 being high quality (≥90% completeness and <5% contamination). Following dereplication at 99% ANI, we generated 146 nonredundant RUG1_ANI99%_ MAGs with 36 high-quality MAGs. The range of their sum genome length was 0.41–5.84‍ ‍Mb, with N50 ranging between 1.66 and 93.2‍ ‍kb (Table S2). A comparison with MAGs in the RUG2 (8,153 genomes) and GTDB (317,542 genomes) databases revealed 16 and 130 RUG1_ANI99%_ MAGs that were potential novel species and strains, respectively (Table S2). Therefore, the RUG2 and GTDB databases did not completely cover the diversity of the rumen microbiota from the tested cattle.

After aligning filtered reads against RUG1_ANI95%_ MAGs, we identified 32 MAGs that were present (relative abundance >0) in >90% of the tested cattle (*n*=20), suggesting that they are core rumen bacteria in Japan (Table S3). The average mapping rate was 43.9% per sample, indicating that RUG1_ANI95%_ MAGs did not cover the full rumen microbial diversity of these animals. This conclusion is also apparent because we did not recover MAGs assigned to core rumen microbial taxa, such as *Fibrobacter*. These MAGs may be unobtainable because we had less data than other studies (Stewart *et al.*, 2019; Anderson and Fernando, 2021).

The 32 and 50 RUG1_ANI99%_ MAGs were classified as members of *Bacteroidota* and *Firmicutes*_A, respectively (Fig. 1 and Table S2); this taxonomic assignment is consistent with that reported in a previous study in which many MAGs reconstructed from the cattle rumen were classified into these phyla (Gharechahi *et al.*, 2021). The remaining RUG1_ANI99%_ MAGs were assigned to the following 12 phyla: *Firmicutes*_C (23 MAGs), *Actinobacteriota* (10 MAGs), *Firmicutes* (9 MAGs), *Verrucomicrobiota* (7 MAGs), *Spirochaetota* (4 MAGs), *Patescibacteria* (3 MAGs), *Proteobacteria* (2 MAGs), *Methanobacteriota* (2 MAGs), *Planctomycetota* (1 MAG), *Synergistota* (1 MAG), *Desulfobacterota*_I (1 MAG), and *Elusimicrobiota* (1 MAG). In *Bacteroidota* MAGs, 10 RUG1_ANI99%_ MAGs were classified as *Prevotella*, which is the most abundant bacterial genus in the rumen (Henderson *et al.*, 2015), and its abundance increases with high-concentrate diets (Luo *et al.*, 2017). Most of the animals tested were offered high-concentrate diets (Table S1), resulting in the reconstruction of *Prevotella* MAGs. In the present study, 14 *Lachnospiraceae* MAGs were reconstructed; however, we previously recovered only 3 *Lachnospiraceae* MAGs (Sato *et al.*, 2021). *Lachnospiraceae* is associated with feed efficiency in ruminants (Li and Guan, 2017) and is crucial for shaping the characteristics of JB rumen microbiomes (Ogata *et al.*, 2019; Sato *et al.*, 2021). Therefore, the *Lachnospiraceae* MAGs obtained herein may be useful for metagenomic and metatranscriptomic research in ruminants, particularly JB cattle.

Prodigal (Hyatt *et al.*, 2010) predicted 330,575 CDS from RUG1_ANI99%_ MAGs, with a range of 537–8,276 (Table S2). We found 156,599 CDS (47.4%) in the KEGG Orthology (KO) database. Consistent with our previous findings (Sato *et al.*, 2021), a KO ana­lysis indicated that most RUG1_ANI99%_ MAGs were roughly clustered according to their phylum (Fig. S1). We focused on genes related to VFA production because VFAs are a major energy source for ruminants (Table 1 and Fig. S2). We identified 113 RUG1_ANI99%_ MAGs (77.4%) with at least one gene related to acetate production (K00925: acetate kinase, K00625: phosphate acetyltransferase). These genes were absent from *Firmicutes*_C RUG1_ANI99%_ MAGs. Rumen propionate is produced via the succinate and‍ ‍acrylate pathways (Jeyanathan *et al.*, 2014). As markers of the succinate pathway, we selected genes encoding methylmalonyl-CoA mutase (K01847, K01848, and K01849), methylmalonyl-CoA epimerase (K05606), and methylmalonyl-CoA decarboxylase (K11264); acrylate pathway markers were genes encoding lactoyl-CoA dehydratase (K20626 and K20627). Succinate pathway marker genes were present in 52 RUG1_ANI99%_ MAGs; most were MAGs belonging to *Bacteroidota* and *Firmicutes*_C, including *Prevotella* and *Succiniclasticum* MAGs (10 and 15 MAGs, respectively). *Prevotella* (Wirth *et al.*, 2018) and *Succiniclasticum* (Van Gylswyk, 1995) both produce propionate from succinate in the rumen. In contrast, none of the RUG1_ANI99%_ MAGs possessed the acrylate pathway marker gene.

Among 146 RUG1_ANI99%_ MAGs, 136 and 112 possessed the genes encoding CAZymes GH13 (α-amylase) and GH77 (4-α-glucanotransferase), respectively, both of which are involved in starch degradation (Fig. S3 and Table S4). Furthermore, oligosaccharide-degrading GH2 (β-galactosidase) and GH3 (β-glucosidase) were present in 92 and 98 RUG1_ANI99%_ MAGs, respectively. GH2, GH3, and GH13 were among the most dominant CAZymes in the cattle rumen (Wang *et al.*, 2019). Additionally, *Prevotella* RUG1_ANI99%_ MAGs had multiple genes related to CAZymes (Fig. S3), which is in accordance with the ability of the genus to degrade fiber sources, such as hemicellulose and pectin (Stewart *et al.*, 1997). All *Prevotella* RUG1_ANI99%_ MAGs possessed genes encoding GH2 (β-galactosidase), GH3 (β-glucosidase), GH5 (endo-β-1,4-glucanase, endo-β-1,4-xylanase, and β-mannosidase), GH13 (α-amylase), GH26 (endo-β-1,4-mannanases), GH32 (invertase), GH36 (α-galactosidase), GH43 (β-xylosidase), GH73 (lysozyme), GH94 (cellobiose phosphorylase), and GH97 (α-glucosidase).

Cellulosomes (Bayer *et al.*, 2004; Artzi *et al.*, 2017) and amylosomes (Ze *et al.*, 2015) consist of scaffolding subunits that contain cohesion modules and dockerin modules, which append to an enzyme for the degradation of lignocellulose and starch, respectively. Cohesin modules specifically interact with dockerin modules, and the cohesin–dockerin interaction is responsible for the integration of enzymes into the complex (Bayer *et al.*, 2004; Artzi *et al.*, 2017). Therefore, we searched cohesin and dockerin domains in RUG1_ANI99%_ MAGs. The proteins containing cohesin and dockerin domains are summarized in Table S5. In total, 200 dockerin and 18 cohesin domains were present in 25 RUG1_ANI99%_ MAGs, including 2 *Ruminococcus* and 11 *Ruminococcus_E* MAGs. Sixteen dockerin-containing proteins carried CAZyme domains, mostly including GH families containing α-amylase (GH13_14, GH13_19, and GH13_28). Among the 25 RUG1_ANI99%_ MAGs, 13 had both cohesin- and dockerin-containing proteins, while 9 had only dockerin-containing proteins. Dassa *et al.* (2014) reported that *Ruminococcus albus* 8, which degrades cellulosic substrates, harbored no cohesion-containing protein. Therefore, we cannot rule out the possibility that RUG1_ANI99%_ MAGs, which had no cohesion- and some dockerin-containing proteins, used an alternative mechanism for the immobilization of dockerin-containing enzymes onto carbohydrates, similar to *Ruminococcus albus* 8 (Dassa *et al.*, 2014).

In *Bacteroidetes*, CAZymes are often organized in PULs. We herein identified 215 PULs in *Bacteroidota* RUG1_ANI99%_ MAGs, and 93 lacked any known CAZyme domains (Table S6). The most common PUL was a single susC/susD-like pair, which was consistent with previous findings (Stewart *et al.*, 2019). Among 32 *Bacteroidota* MAGs, 29 had at least one PUL. The number of PULs per *Bacteroidota* MAG ranged between 1 and 18, with the highest number being observed in *Prevotella* MAG and *Cryptobacteroides* MAG (JB_Ai_91_bin_120 and F1_Ai_424_bin_68, respectively). Ninety-eight and 80 PULs were identified in *Cryptobacteroides* MAG and *Prevotella* MAGs, respectively, and contained various CAZymes. Overall, the present results suggest that *Cryptobacteroides* and *Prevotella* are important for rumen function (specifically carbon degradation) in cattle in Japan.

In summary, we reconstructed 146 rumen-specific MAGs from cattle in Japan, with many being potentially novel. These MAGs are valuable resources for enhancing the resolution of future metagenome- and metatranscriptomic-based taxonomical and functional studies.

## Citation

Sato, Y., Takebe, H., Oishi, K., Yasuda, J., Kumagai, H., Hirooka, H., and Yoshida, T. (2022) Identification of 146 Metagenome-assembled Genomes from the Rumen Microbiome of Cattle in Japan. *Microbes Environ ***37**: ME22039.

https://doi.org/10.1264/jsme2.ME22039

## Supplementary Material

Supplementary Material 1

Supplementary Material 2

Supplementary Material 3

Supplementary Material 4

Supplementary Material 5

Supplementary Material 6

Supplementary Material 7

## Figures and Tables

**Fig. 1.**
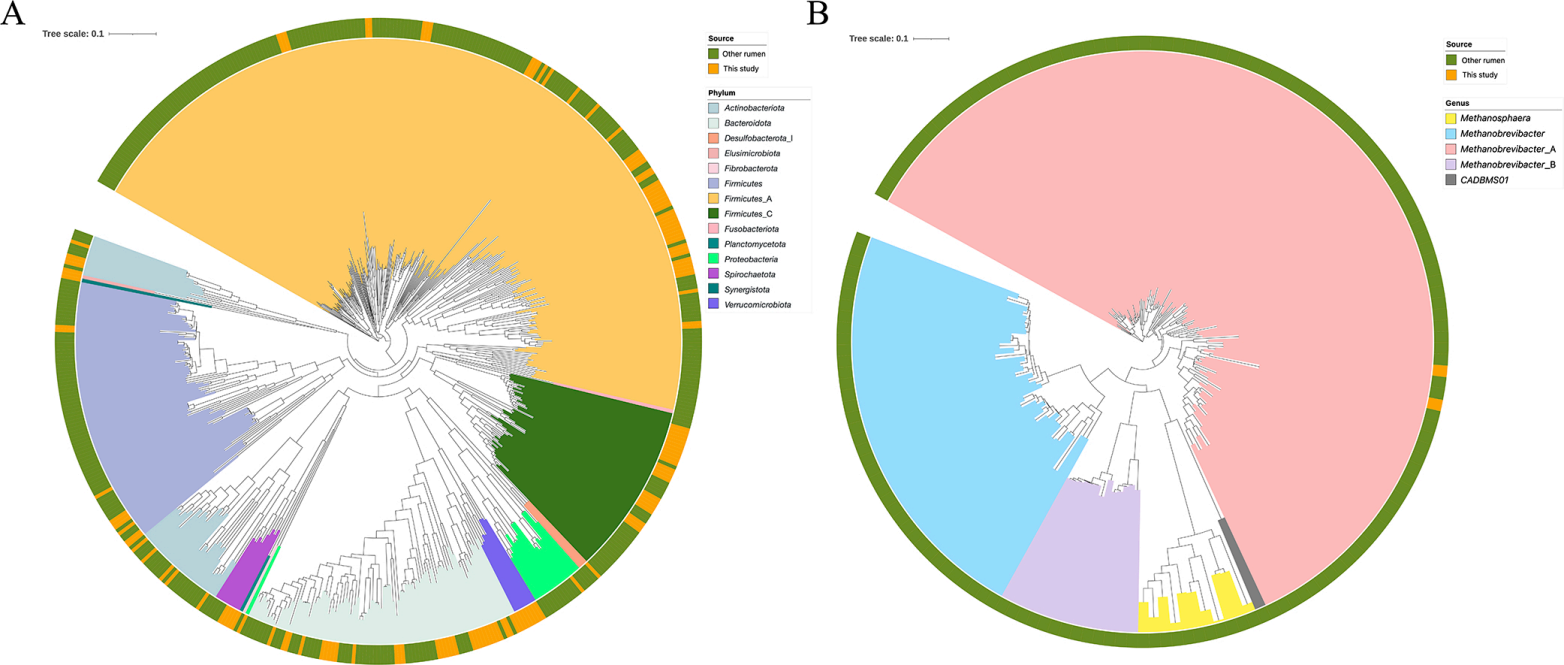
Phylogenetic tree of metagenomic-assembled genomes (MAGs). (A) Bacterial and (B) archaeal phylogenetic trees of RUG1_ANI99%_ MAGs. The bacterial tree includes the bacterial genomes of Hungate1000 (Seshadri *et al.*, 2018), and the archaeal tree contains previously reported *Methanobacteriota* genomes (Seshadri *et al.*, 2018; Stewart *et al.*, 2019; Anderson and Fernando, 2021). Visualization was performed in iTOL (Letunic and Bork, 2021). The outer ring indicates the genome source. Three MAGs (JB_Iw_11_bin_33, JB_Ai_53_bin_86, and merge_bin_133) were excluded because they did not contain enough marker genes.

**Table 1.** The number of RUG1_ANI99%_ MAGs associated with volatile fatty acid production

Phylum^1^	Acetate^2^	Propionate^3^		Butyrate^4^
		Succinate pathway	Acrylate pathway	
*Actinobacteriota* (10)	9	2	0	4
*Bacteroidota* (32)	29	22	0	15
*Desulfobacterota*_I (1)	1	0	0	0
*Elusimicrobiota* (1)	1	0	0	1
*Firmicutes* (9)	8	0	0	4
*Firmicutes*_A (50)	48	4	0	24
*Firmicutes*_C (23)	0	23	0	22
*Patescibacteria* (3)	3	0	0	0
*Planctomycetota* (1)	1	0	0	0
*Proteobacteria* (2)	2	0	0	1
*Spirochaetota* (4)	4	0	0	1
*Synergistota* (1)	0	1	0	1
*Verrucomicrobiota* (7)	7	0	0	2
*Methanobacteriota* (2)	0	0	0	2

^1^ The number after the phylum name shows MAG numbers of the phylum in RUG1_ANI99%_ MAGs. ^2^ Numbers of MAGs that have genes assigned as K00925 and/or K00625. ^3^ Numbers of MAGs that possessed at least one succinate pathway marker gene (K01847, K01848, K01849, K11264, and K05606) or acrylate pathway marker gene (K20626 and K20627). ^4^ Numbers of MAGs with at least one gene related to butyrate production (K00634, K00929, K01034, K00626, and K00074).
