# Supplementary material for: Identification of 146 Metagenome-assembled Genomes from the Rumen Microbiome of Cattle in Japan
Source: Microbes Environ. 2022 Oct 21;37(4):ME22039. doi: 10.1264/jsme2.ME22039 (PMC9763041; doi:10.1264/jsme2.ME22039)
Supplement: Supplementary file 1 — Supplementary Material 1 [file 37_22039_s1.pdf]

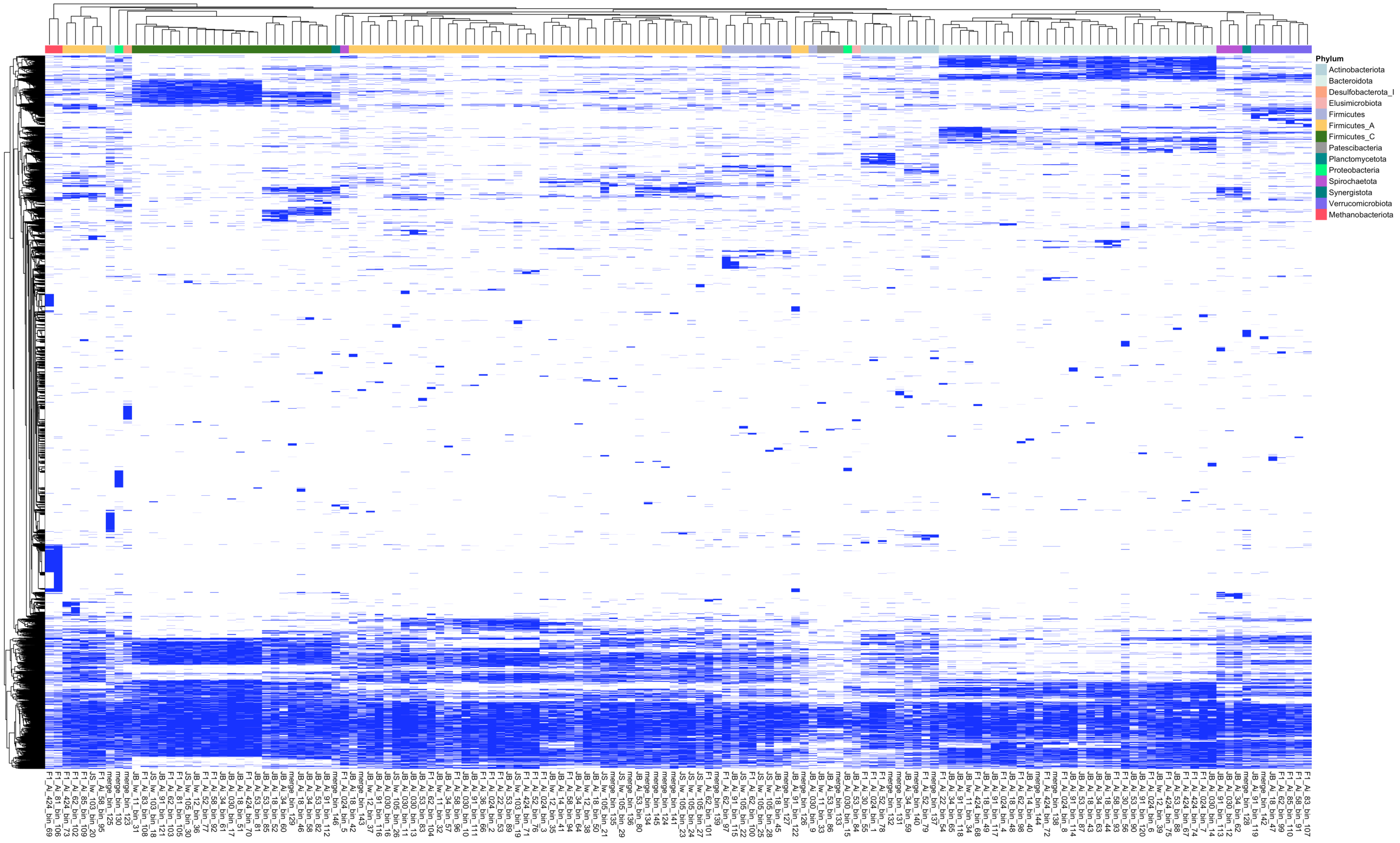


**Fig. S1 Binary heatmap of KEGG orthology (KO) found in the rumen metagenomic-assembled genomes (MAGs).** Each row indicates one KO and each column represents one MAG. Presence is depicted in blue, and absence is depicted in white.

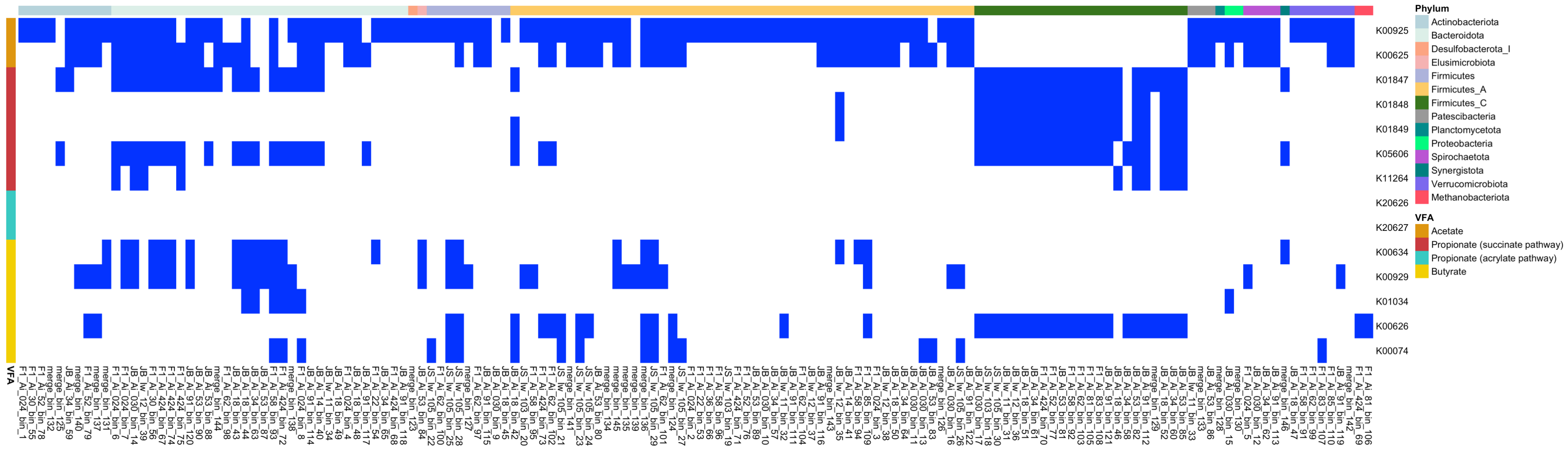


**Fig. S2 Binary heatmap of KEGG orthology (KO) results related to the production of volatile fatty acids (VFAs) in rumen metagenomic-assembled genomes (MAGs).** Each row indicates one KO and each column represents one MAG. Blue indicates presence and white indicates absence.

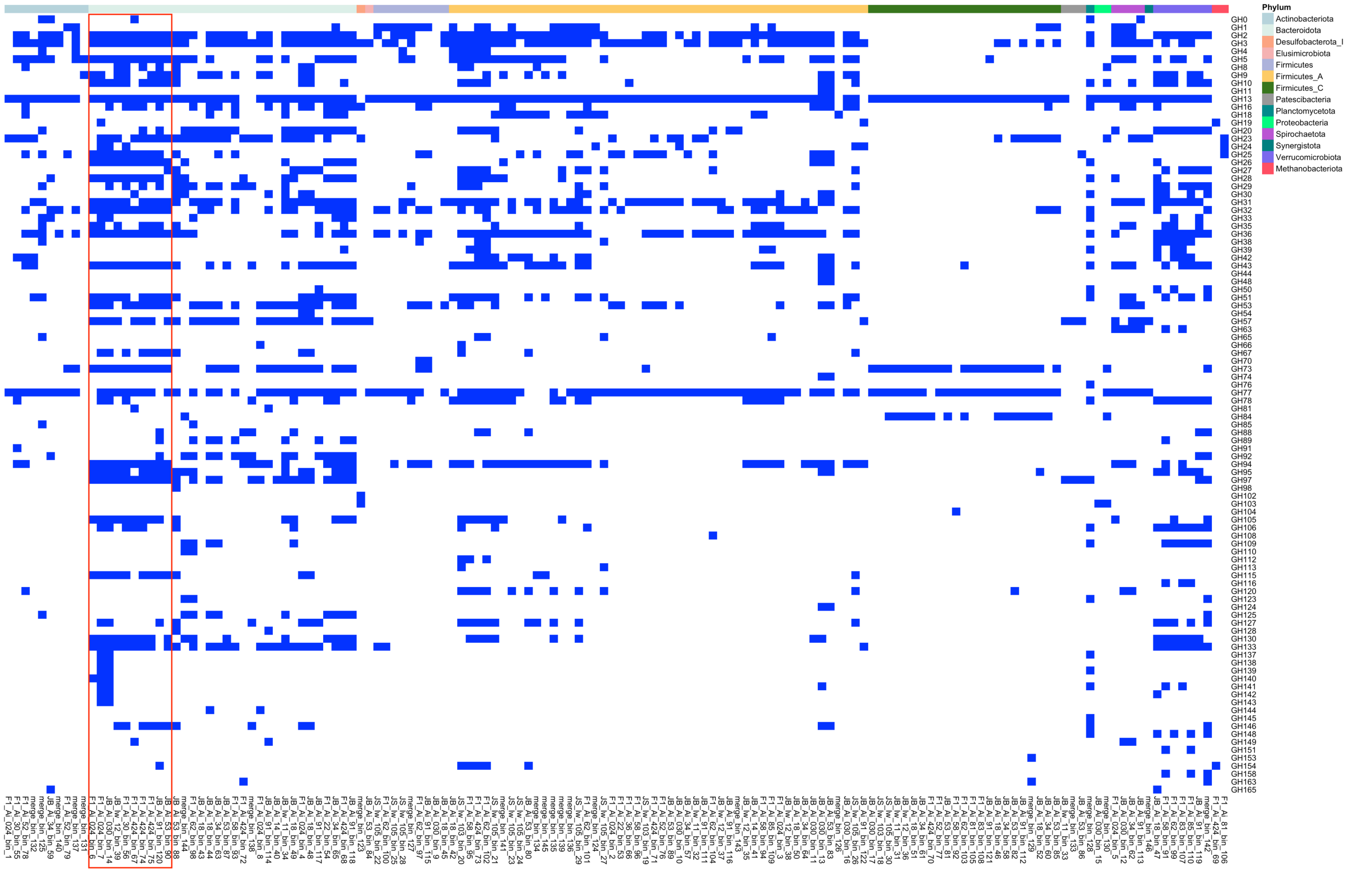


**Fig. S3 Binary heatmap of glycoside hydrolases (GH) in rumen metagenomic-assembled genomes (MAGs).** The red box indicates *Prevotella* MAGs. Each row is one GH family, and each column is one MAG. Blue indicates presence and white indicates absence.
